# Mitochondrial Respiratory Chain Inhibitors Involved in ROS Production Induced by Acute High Concentrations of Iodide and the Effects of SOD as a Protective Factor

**DOI:** 10.1155/2015/217670

**Published:** 2015-07-29

**Authors:** Lingyan Wang, Qi Duan, Tingting Wang, Mohamed Ahmed, Na Zhang, Yongmei Li, Lanying Li, Xiaomei Yao

**Affiliations:** ^1^Department of Pathophysiology, School of Basic Medical Sciences, Tianjin Medical University, Tianjin 300070, China; ^2^Key Lab of Hormones and Development Ministry of Health, Institute of Endocrinology, Metabolic Disease Hospital, Tianjin Medical University, Tianjin 300070, China

## Abstract

A major source of reactive oxygen species (ROS) generation is the mitochondria. By using flow cytometry of the mitochondrial fluorescent probe, MitoSOX Red, western blot of mitochondrial ROS scavenger Peroxiredoxin (Prx) 3 and fluorescence immunostaining, ELISA of cleaved caspases 3 and 9, and TUNEL staining, we demonstrated that exposure to 100 *μ*M KI for 2 hours significantly increased mitochondrial superoxide production and Prx 3 protein expression with increased expressions of cleaved caspases 3 and 9. Besides, we indicated that superoxide dismutase (SOD) at 1000 unit/mL attenuated the increase in mitochondrial superoxide production, Prx 3 protein expression, and lactate dehydrogenase (LDH) release and improved the relative cell viability at 100 *μ*M KI exposure. However, SOD inhibitor diethyldithiocarbamic acid (DETC) (2 mM), Rotenone (0.5 *μ*M), a mitochondrial complex I inhibitor, and Antimycin A (10 *μ*M), a complex III inhibitor, caused an increase in mitochondrial superoxide production, Prx 3 protein expression, and LDH release and decreased the relative cell viability. We conclude that the inhibitors of mitochondrial respiratory chain complex I or III may be involved in oxidative stress caused by elevated concentrations of iodide, and SOD demonstrates its protective effect on the Fischer rat thyroid cell line (FRTL) cells.

## 1. Introduction

Reactive oxygen species (ROS) are required for normal physiologic function and are linked to thyroid hormone synthesis, yet they become toxic when produced in excessive amounts [1]. From the previous works, we found that elevated concentrations of iodide instigated oxidative stress, which had time-course and concentration response [2]. Adequate iodide intake is required for normal thyroid function, but ROS production due to the elevated concentrations of iodide may disturb the redox equilibrium and may become toxic for intracellular macromolecules, such as DNA, proteins, lipids, and nucleic acids [1–5].

Thyroid hormone receptors have been detected in mitochondria which are involved in the regulation of energy mechanism and apoptosis [6]. One of the major sources of ROS are mitochondria, especially superoxide anions, which are highly involved in mitochondrial dysfunction [6, 7]. Increased levels of ROS may activate the mitochondrial cascade of apoptosis [8, 9]. The predominant source of ROS production is mitochondrial respiration [10]. After the experiments done by Chance [11, 12], two main sites that produce these superoxides have been reported: mitochondrial complex I and complex III [13–15]. Reducing NADH dehydrogenase within complex I results in superoxide radical production. Complex III can generate ROS and release ROS into the inner membrane space or mitochondrial matrix [14–16].

Thyrocytes have antioxidant mechanisms which include superoxide dismutases (SOD) and Peroxiredoxins (Prxs) that contribute to limiting cellular injuries [17]. Several pathways exist within the mitochondria to detoxify superoxide anions. The initial detoxification step is to convert superoxide anions into hydrogen peroxide (H_2_O_2_) by SOD [17–19]. Prx 3 can be the target for up to 90% of hydrogen peroxide generated in the mitochondrial matrix [20].

Apoptosis is commonly instigated via two main pathways: extrinsic pathway which is induced by death receptor and intrinsic pathway which is mediated by mitochondria [21, 22]. Mitochondrial-mediated apoptotic intrinsic pathway is started in reaction to cellular damages, and damaged mitochondria may release cytochrome c and activate a series of caspases [23, 24]. Caspase activation is the final process of the death signaling pathway, in which procaspase 3 is activated into cleaved caspase 3 following the autoactivation of procaspase 9 to cleaved caspase 9 because of the mitochondrial release of cytochrome c [25], which then comes to apoptosis [26, 27]. A variety of occurrences such as holes, tears, or pores in the outer mitochondrial membrane may lead to the release of cytochrome c [28]. This release often leads to oxidative phosphorylation loss and ROS production [29]. However, the nature of excess iodide induced oxidative stress in mitochondria remains to be determined.

Novelty of the present study is that Rotenone has been commonly used as plant insecticides, Antimycin A has been frequently used as a piscicide for fish culture and may be transmitted to humans by consumption of plants or fish and may present toxicity in humans. Rotenone (0.5 *μ*M) and Antimycin A (10 *μ*M), inhibitors of mitochondrial complex I and complex III, respectively, were used in the present study. SOD are important antioxidant in the thyroids. In order to figure out whether SOD protects against elevated concentrations of iodide induced mitochondrial oxidative stress, counterevidence of SOD inhibitor diethyldithiocarbamic acid (DETC) on the basis of what we have done previously was shown as a contrast. Prx 3 is predominantly located in mitochondria. It plays a key role in mitochondrial antioxidant defense. We demonstrated in the present study that elevated concentrations of KI exposure for 2 hours significantly increased the production of mitochondrial superoxide and the expression of Prx 3 protein in Fischer rat thyroid cell line (FRTL), with increased levels of cleaved caspases 3 and 9. SOD protected against elevated concentrations of iodide instigated the production of mitochondrial superoxide and Prx 3 protein expression, and alleviated apoptosis in FRTL cells. SOD inhibitors and inhibitors of mitochondrial complexes I and III aggravated the production of mitochondrial superoxide, expression of Prx 3 protein, and apoptosis instigated by elevated concentrations of iodide in FRTL cells. Similar changes were observed in triple fluorescence staining and TUNEL staining.

## 2. Material and Methods

### 2.1. Reagents

Rotenone, Antimycin A, goat anti-mouse IgG-HRP, and goat anti-mouse IgG-FITC were bought from Santa Cruz (Santa Cruz Biotechnology, Inc., CA, USA). Anti-Peroxiredoxin-3 antibody was purchased from Abcam (Abcam, Cambridge, MA, USA). Superoxide dismutase (SOD) was bought from Sigma (Sigma-Aldrich, MO, USA). Rat cleaved caspases 3 and 9 ELISA kits were purchased from Chenglin (Chenglin Biotechnology, Beijing, China). TUNEL assay kit was purchased from Boster (Boster, Wuhan, China). FRTL, diethyldithiocarbamate (DETC), MitoSOX Red, MTT, LDH kit, and thyroid stimulating hormone (TSH) were purchased as previously described [2].

### 2.2. Cell Culture

FRTL cells culture method was according to the previous report [2]. FRTL cells were exposed to 100 *μ*M potassium iodide (KI), 2 mM DETC, 100 *μ*M KI + 2 mM DETC, 1000 unit/mL SOD, 100 *μ*M KI + 1000 unit/mL SOD, 0.5 *μ*M Rotenone, 100 *μ*M KI + 0.5 *μ*M Rotenone, 10 *μ*M Antimycin A, and 100 *μ*M KI + 10 *μ*M Antimycin A, respectively for 2 h.

### 2.3. Cell Viability Assay

We tested cell viability with the 3-(4,5-dimethylthiazol-2-yl)-2,5-diphenyltetrazolium bromide (MTT) assay. 10 *μ*L MTT (5 mg/mL) was added in dark and covered with aluminum foil. After incubation for 4 h, we added 100 *μ*L DMSO into each well to dissolve the formazan crystals. We then measured the absorbance at 490 nm by a spectrophotometer (Wallac 1420 VICTOR3, PerkinElmer).

### 2.4. Lactate Dehydrogenase (LDH) Assay

After the different treatment was applied, LDH release in the supernatant was measured employing LDH detection kit. The experiment is established by reducing 2-p-iodophenyl-3-nitrophenyl tetrazolium chloride (tetrazolium INT) to a red formazan which is specifically detected by colorimetric (450 nm) assay.

### 2.5. Flow Cytometry

Flow cytometry was used to measure MitoSOX and analyze the production of mitochondrial superoxide. Cell density was adjusted to 5 × 10^6^ cells/L. The fluorescence intensity of MitoSOX was detected by FACSCalibur (BD Bioscience, San Jose, CA), at the wavelength of 488 nm (excitation)/575 nm (emission). FL2 channel forward scattering (forward scatter, FSC) and lateral scattering (side scatter, SSC) data was then collected. 10000 cells were collected for each sample. The control group without MitoSOX was regarded as the blank zero group for standardization.

### 2.6. Fluorescence Immunostaining

Primary antibody was  Anti-Prx 3 antibody 1 : 1000. Secondary antibodies linked to fluorophores are goat anti-mouse IgG-FITC 1 : 100. MitoSOX Red was used to detect mitochondrial superoxide production. Cells were then incubated with Hoechst 33258 before image acquisition. Fluorescent images were acquired by Zeiss LSM 510 laser confocal microscope (Zeiss, Germany). Immunofluorescence intensity of Prx 3 and MitoSOX were analyzed by Image J software (NIH). Average immunofluorescence intensity was used for statistical analysis.

### 2.7. Western Blot

A bicinchoninic acid (BCA) protein assay kit was used to examine whole cell proteins extracted from different treatment and control groups. 50 *μ*g of protein was transferred to nitrocellulose membrane. The membrane was then incubated overnight with anti-Peroxiredoxin-3 antibody, developed by chemiluminescent substrate. Blots were analyzed by Image J. Intensities of all the blots were normalized with those of *β*-actin.

### 2.8. ELISA of Cleaved Caspases 3 and 9

Cleaved caspases 3 and 9 in the supernatant of the cell cultures following different treatment were measured. ELISA assays were conducted following the instruction of the manufacturer's protocols. The relative amount of cleaved caspases 3 and 9 was determined at a wavelength of 450 nm by spectrophotometry (Wallac 1420 VICTOR3, PerkinElmer). The concentrations of cleaved caspases 3 and 9 in the samples were analyzed according to the standard curve (*n* = 6).

### 2.9. TUNEL Staining

TUNEL staining kit was used to quantify the number of apoptotic cells. 5 × 10^5^ cells were grown on coverslips for each treatment group. After exposure for 2 h, 4% paraformaldehyde solution was used to fix the cells for 30 minutes, TUNEL staining was followed. The positive cells were counted under microscopy (Leika, Germany) at 400x magnification by a blind manner, and five independent fields were randomly selected for each coverslip. Apoptotic cells (%) = TUNEL positive cells/total cells × 100.

### 2.10. Statistics

Data was analyzed by SPSS 17.0 and shown as the mean ± SD. Kolmogorov-Smirnov (K-S) was used for normality test and Levene statistic was used for homogeneity of variances test; least significant difference (LSD) test of one-way analysis of variance (one-way ANOVA) was used for detecting the differences between groups. Statistical significance was considered when *P* < 0.05.

## 3. Results

### 3.1. Determination of the Optimal Concentrations of SOD, Rotenone, and Antimycin A

**Figure 1:**
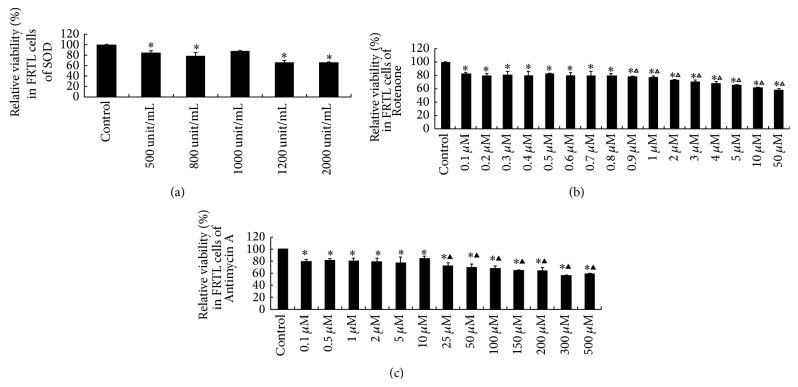
Relative cell viability was tested in FRTL cells after exposure to SOD, Rotenone, and Antimycin A for 2 h to choose the optimal concentrations. (a) Relative viability for the optimal concentration of SOD. (b) Relative viability for the optimal concentration of Rotenone. (c) Relative viability for the optimal concentration of Antimycin A. Data were analyzed by mean ± SD (*n* = 6). ^*∗*^ *P* < 0.05 versus control group. ^△^ *P* < 0.05 versus 0.5 *μ*M rotenone group. ^▲^ *P* < 0.05 versus 10 *μ*M Antimycin A group.

The optimal concentrations of SOD, Rotenone, and Antimycin A used in the present study were determined by the result of relative cell viability following the treatment of 500, 800, 1000, 1200, and 2000 unit/mL SOD (Figure 1(a)), 0.1, 0.2, 0.3, 0.4, 0.5, 0.6, 0.7, 0.8, 0.9, 1, 2, 3, 4, 5, 10, and 50 *μ*M Rotenone (Figure 1(b)), and 0.1, 0.5, 1, 2, 5, 10, 25, 50, 100, 150, 200, 300, and 500 *μ*M Antimycin A (Figure 1(c)) to FRTL cells for 2 hours. Compared to the control group, no significant change of relative cell viability was found in 1000 unit/mL SOD group, while 500, 800, 1200, and 2000 unit/mL SOD significantly decreased the relative viability (*P* < 0.05) (Figure 1(a)). Compared to the control group, although all the relative cell viability was decreased following Rotenone exposure (*P* < 0.05), when compared to the 0.5 *μ*M Rotenone group, the relative viability was significantly decreased when the concentrations of Rotenone reach above 0.9 *μ*M (*P* < 0.05), although there were not any significant differences that can be detected in 0.1, 0.2, 0.3, 0.4, 0.6, 0.7, and 0.8 *μ*M Rotenone groups (*P* > 0.05) (Figure 1(b)). 0.1, 0.5, 1, 2, 5, 10, 25, 50, 100, 150, 200, 300, and 500 *μ*M Antimycin A decreased the relative cell viability when compared to the control group (*P* < 0.05). Compared to the 10 *μ*M Antimycin A group, despite the fact that no significant differences of relative viability were detected in 0.1, 0.5, 1, 2, and 5 *μ*M groups, 25, 50, 100, 150, 200, 300, and 500 *μ*M Antimycin A treatment groups showed significantly decreased relative viability (*P* < 0.05) (Figure 1(c)). Based on the dose response of relative viability, we choose 1000 unit/mL SOD, 0.5 *μ*M Rotenone, and 10 *μ*M Antimycin A as the optimal concentrations for FRTL cells in the present study.

### 3.2. Effects of DETC, SOD, Rotenone, and Antimycin A on Elevated Iodide Instigated Relative Viability

**Figure 2:**
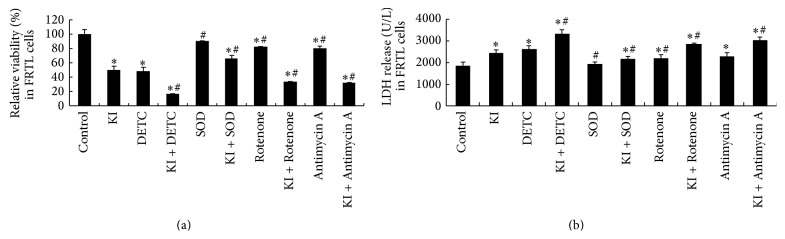
Relative viability and LDH release were detected in FRTL cells following 100 *μ*M KI, DETC, SOD, Rotenone, and Antimycin A exposure for 2 h. (a) Effect of DETC, SOD, Rotenone, and Antimycin A on 100 *μ*M KI exposure for 2 h induced relative viability in FRTL cells. (b) Effect of DETC, SOD, Rotenone, and Antimycin A on 100 *μ*M KI exposure for 2 h induced LDH release in FRTL cells. Data were analyzed by mean ± SD (*n* = 6). ^*∗*^ *P* < 0.05 versus control group. ^#^ *P* < 0.05 versus KI group.

Relative viability of the 100 *μ*M KI group was decreased significantly at 2 h when compared to the control group (*P* < 0.05). Compared to the 100 *μ*M KI group, an increase of relative cell viability was detected in SOD, KI + SOD group, in Rotenone group, and in Antimycin A group (*P* < 0.05). However, a significant decrease was detected in KI + DETC group, KI + Rotenone group, and in KI + Antimycin A group (*P* < 0.05). We suggest that the decreased relative cell viability instigated by KI (100 *μ*M) could be improved by SOD (1000 unit/mL). However, DETC, Rotenone, or Antimycin A can further decrease the relative cell viability instigated by KI in FRTL cells (Figure 2(a)).

### 3.3. Effects of DETC, SOD, Rotenone, and Antimycin A on Elevated Iodide Instigated LDH Release

Significant changes were found in LDH release detection. The LDH release of KI exposure group was significantly increased at 2 h when compared with the control group (*P* < 0.05). We discovered that a significant decrease of LDH release was detected in SOD and in KI + SOD group when compared to KI group (*P* < 0.05), while the increased LDH release instigated by KI (100 *μ*M) could be further increased by DETC, Rotenone, and Antimycin A in FRTL cells (Figure 2(b)), with significant increase being observed in KI + DETC group, KI + Rotenone group, and KI + Antimycin A group (*P* < 0.05).

### 3.4. Effects of DETC, SOD, Rotenone, and Antimycin A on Elevated Iodide Instigated the Production of Mitochondrial Superoxide

After mitochondrial superoxide production was measured, we found that, except for the SOD treatment group, all the other treatment groups were found significantly increased at 2 h compared to the control group (*P* < 0.05).

**Figure 3:**
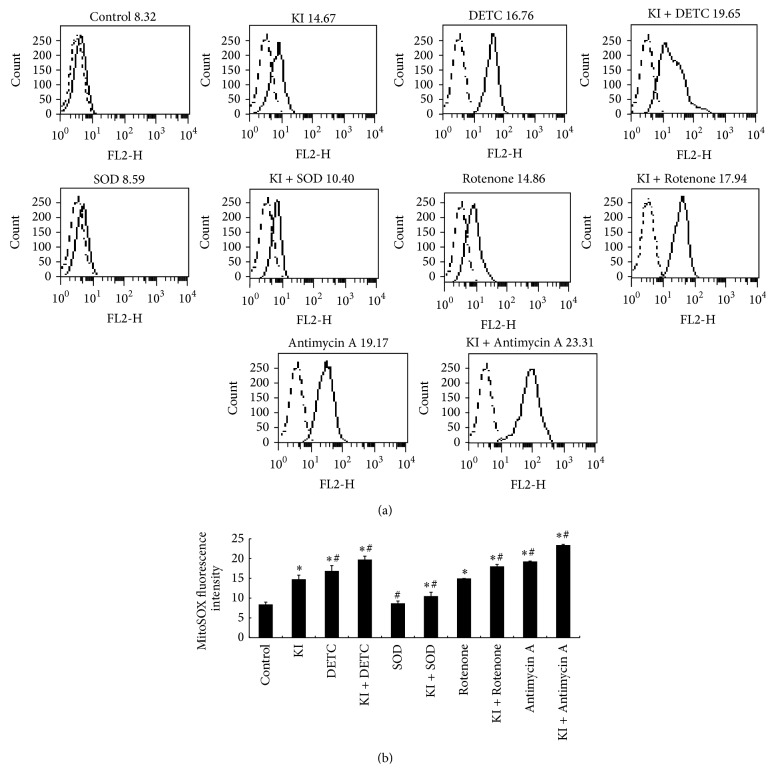
Effect of DETC, SOD, Rotenone, and Antimycin A treatment for 2 h on KI-instigated production of mitochondrial superoxide in FRTL cells. (a) Representative histograms of the mean fluorescence intensity of MitoSOX Red. (b) The production of mitochondrial superoxide instigated by KI in FRTL cells could be increased by DETC, Rotenone, and Antimycin A but decreased by SOD. Dotted line represents the histogram analysis of the blank zero group, the solid line represents the control group or the different treatment groups, respectively. Data were analyzed by mean ± SD. ^*∗*^ *P* < 0.05 versus control group. ^#^ *P* < 0.05 versus KI group.

We demonstrated that significant decrease of mitochondrial superoxide production was detected in SOD group, as well as in the KI and SOD treatment group when compared to the KI group (*P* < 0.05), suggesting that the increased production of mitochondrial superoxide instigated by KI (100 *μ*M) could be decreased by SOD (1000 unit/mL). At the same time, a significant increase was found in DETC group, KI + DETC group, KI + Rotenone group, Antimycin A group, and KI + Antimycin A group (*P* < 0.05). Similar changes in fluorescence staining of MitoSOX following the treatment of DETC, SOD, Rotenone, or Antimycin A were observed (Figures 5(a) and 5(b)). We suggest that DETC, Rotenone, or Antimycin A can further increase the production of mitochondrial superoxide instigated by KI (100 *μ*M) in FRTL cells (Figure 3).

### 3.5. Effect of DETC, SOD, Rotenone, or Antimycin A on KI Induced Prx 3 Expression

**Figure 4:**
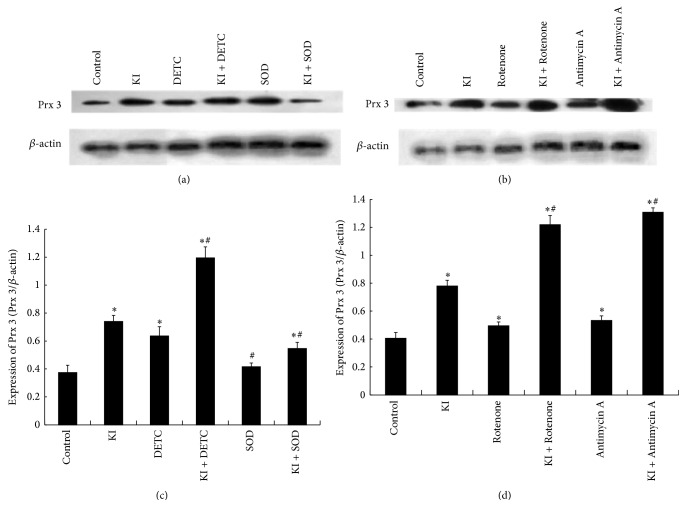
Densitometric analysis showed effects of DETC, SOD, Rotenone, or Antimycin A on KI induced Prx 3 expression. (a, b) Representative western blot of Prx 3 (27 kDa) and *β*-actin (45 kDa). (c) Western blot analysis of DETC, SOD on KI induced Prx 3 expression. (d) Western blot analysis of Rotenone, Antimycin A on KI induced Prx 3 expression. Values of Prx 3 and *β*-actin expression obtained from Image J were used for relative density calculation and normalization (c, d). Data were analyzed by mean ± SD (*n* = 6). ^*∗*^ *P* < 0.05 versus control group. ^#^ *P* < 0.05 versus KI group.

**Figure 5:**
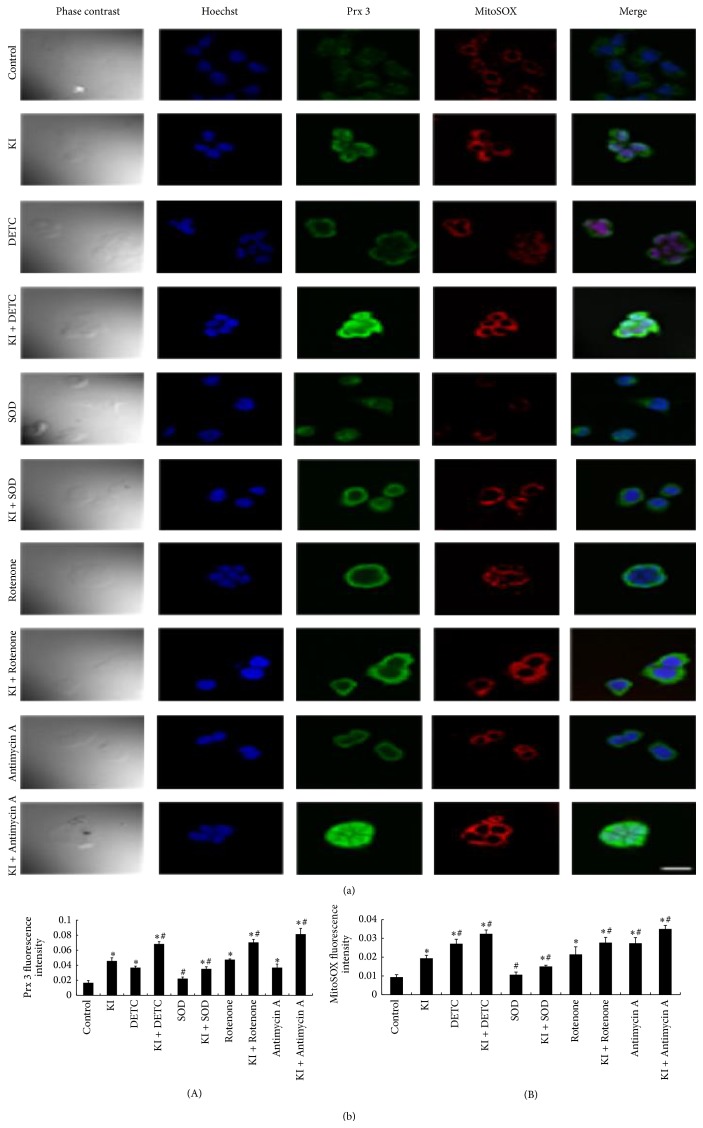
(a) Changes in immunofluorescence of MitoSOX and Prx 3 after the treatment of DETC, SOD, Rotenone, and Antimycin A. The cells were stained with specific antibodies of Prx 3 protein (green) and incubation with MitoSOX (red); nucleus was dyed with Hoechst (blue). Scale bar = 50 *μ*m. (b) Prx 3 and MitoSOX fluorescence intensity (A, B) under different treatment. Data were analyzed by mean ± SD (*n* = 6). ^*∗*^ *P* < 0.05 versus control group. ^#^ *P* < 0.05 versus KI group.

The expression of Prx 3 in the KI group, DETC group, KI + DETC group, KI + SOD group, Rotenone group, KI + Rotenone group, Antimycin A group, and KI + Antimycin A group was significantly increased at 2 h when compared to the control group (*P* < 0.05). The increased expression of Prx 3 instigated by KI was significantly decreased by SOD group, KI + SOD group (*P* < 0.05). However, it increased by KI + DETC group, KI + Rotenone group, and KI + Antimycin A group (*P* < 0.05) (Figure 4). Similar changes can be observed in immunofluorescence staining of Prx 3 following the treatment of DETC, SOD, Rotenone, or Antimycin A (Figures 5(a) and 5(b)).

### 3.6. Changes in Immunofluorescence Staining of MitoSOX and Prx 3 following the Treatment of DETC, SOD, Rotenone, or Antimycin A

100% of fluorescent cells in each focus had been analyzed, which could be seen in the phase contrast picture. When compared to the control group, strong fluorescence of both Prx 3 and MitoSOX can be observed in 2 h KI exposure group. When compared with the KI group, a weaker fluorescence of both Prx 3 and MitoSOX was found in SOD group, KI + SOD group (*P* < 0.05), suggesting that the increased fluorescence intensity of Prx 3 and MitoSOX instigated by KI (100 *μ*M) could be suppressed by SOD (1000 unit/mL) treatment. However, a much stronger fluorescence of both Prx 3 and MitoSOX was noticed in KI + DETC group, KI + Rotenone group, KI + Antimycin A group (*P* < 0.05) (Figures 5(a) and 5(b)), which indicated that the increased fluorescence of Prx 3 and MitoSOX instigated by KI can further be enhanced by DETC, Rotenone, or Antimycin A treatment in FRTL cells (Figures 5(a) and 5(b)). Consistently, these changes in immunofluorescence staining were verified by flow cytometry (Figure 3) and western blot (Figure 4).

### 3.7. Changes in Cleaved Caspases 3 and 9 following the Treatment of DETC, SOD, Rotenone, or Antimycin A

**Figure 6:**
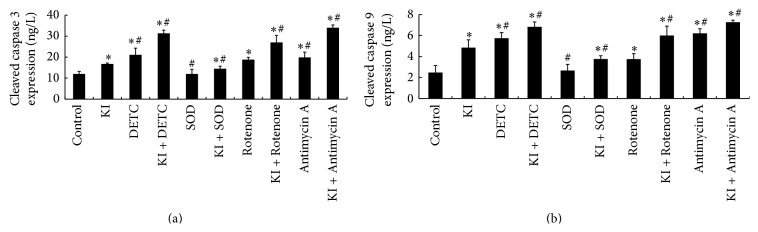
Cleaved caspases 3 and 9 levels were measured following DETC, SOD, Rotenone, and Antimycin A exposure for 2 h by ELISA. (a) Changes of cleaved caspase 3 following DETC, SOD, Rotenone, or Antimycin A treatment in FRTL cells. (b) Changes of cleaved caspase 9 following DETC, SOD, Rotenone, or Antimycin A treatment in FRTL cells. Data were analyzed by mean ± SD (*n* = 6). ^*∗*^ *P* < 0.05 versus control group. ^#^ *P* < 0.05 versus KI group.

Changes of cleaved caspases 3 and 9 were measured in the supernatant of the culture. Analysed from the results, we indicated that KI group induced the cleaved caspases 3 and 9 expression increased at 2 h when compared to the control group (*P* < 0.05). Compared to the KI group, a significant decrease of cleaved caspase 3 and 9 was detected in the SOD and KI + SOD groups, suggesting that the increased cleaved caspases 3 and 9 instigated by KI (100 *μ*M) can be decreased by SOD (1000 unit/mL) (*P* < 0.05). However, the increased cleaved caspases 3 and 9 induced by KI (100 *μ*M) can further be increased by DETC, Rotenone, or Antimycin A treatment, with the results of a significant increase of cleaved caspases 3 and 9 expression being detected in the KI + DETC group, KI + Rotenone group, and in KI + Antimycin A group (*P* < 0.05) (Figure 6).

### 3.8. Changes in Apoptotic Cells following the Treatment of DETC, SOD, Rotenone, or Antimycin A

**Figure 7:**
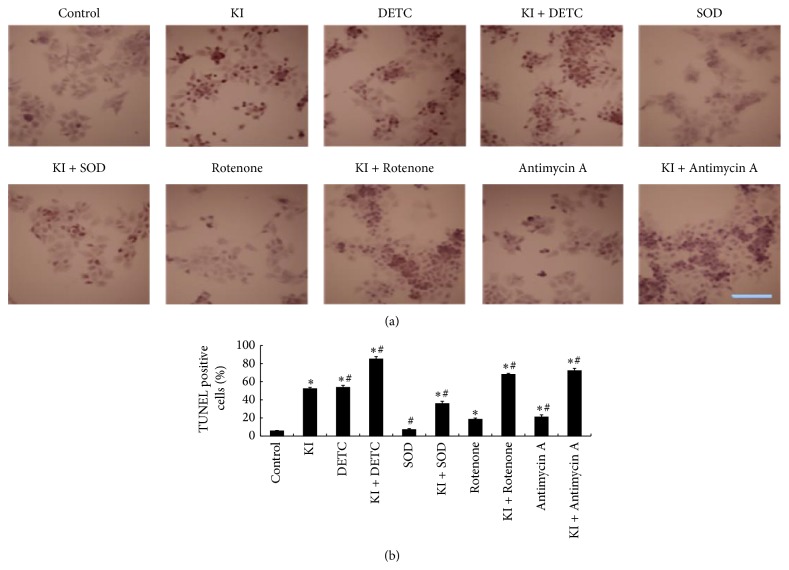
TUNEL positive cells were detected following DETC, SOD, Rotenone, or Antimycin A treatment for 2 h in FRTL cells. (a) TUNEL-positive cells under microscopy (400x light microscopy). (b) Representative of percentage of TUNEL positive cells. Nucleus of apoptotic cells was dyed with TUNEL (Brown). Scale bar = 50 *μ*m. Data were analyzed by mean ± SD (*n* = 6). ^*∗*^ *P* < 0.05 versus control group. ^#^ *P* < 0.05 versus KI group.

TUNEL positive cells were counted in a blind manner under light microscopy at 400x magnification from at least three independent fields. Percentage of apoptotic cells was calculated. Compared with the control group, the apoptotic cells (%) of KI group were significantly increased at 2 h (*P* < 0.05), indicating KI (100 *μ*M) induced cell apoptosis. To study the effect of SOD, DETC, Rotenone, and Antimycin A on KI (100 *μ*M) induced cell apoptosis, we found that the apoptotic cells (%) were decreased in SOD and in KI + SOD groups when compared to the KI group (*P* < 0.05). However, the KI + DETC groups, KI + Rotenone group, or KI + Antimycin A did exhibit significant increases (*P* < 0.05). We indicated that the cell apoptosis instigated by KI (100 *μ*M) can be decreased by SOD (1000 unit/mL) and can be further increased by DETC, Rotenone, or Antimycin A in FRTL cells (Figure 7).

## 4. Discussion

In the current study, we demonstrated that the effects of SOD, DETC (an inhibitor of SOD), Rotenone, which is a mitochondrial complex I inhibitor, and Antimycin A, a complex III inhibitor on 100 *μ*M KI exposure for 2 h, induced the production of mitochondrial superoxide and the expression of Prx 3 protein in FRTL cells. One of the principal sources of oxidative stress is mitochondrial superoxide production. In addition, potent producers of superoxide anions are mitochondrial complexes I and III of the electron transport chain [30]. By concentrating on elevated concentrations of iodide instigating the production of superoxide and targeting the mitochondrial electron transport chain by using SOD, SOD inhibitors, and mitochondrial complex I or III inhibitors, we aimed to figure out the mitochondrial oxidant/antioxidant related mechanisms underlying potential thyroid disorders [31].

The inhibitors of complex I (Rotenone) and complex III (Antimycin A) have been commonly used. But the concentration being used is not stationary, causing experiment methods to be different from each other. Bongard et al. used 20 *μ*M rotenone as complex I inhibitor to perfuse an isolated intact rat lung to test energy charge and pulmonary endothelial permeability [32]. Cillero-Pastor et al. used 10 and 50 *μ*g/mL Rotenone to culture human chondrocytes, to investigate mRNA expression [33]. 100 nM Rotenone was treated to hypoglycemic cells to express the inhibitory effect [34]. There is ROS production in the presence of Rotenone when digitonin protein solubilizates with glycerol-3-phosphate (GP) [35]. Cyclic electron transport activity can be inhibited by Antimycin A [36]. Experiment of concentrations of 0 to 12 mM Antimycin A was designed to examine the effect of inhibition of Antimycin A [37]. 1 *μ*g/mL Antimycin A or 12 *μ*M CoQ1 was added in the medium for the detection of hydrogen peroxide production [35]. 30 *μ*M Rotenone and Antimycin A inhibit the electricity outputs in* Candida melibiosica* yeast-based biofuel cell [38]. By using the measurements of mitochondrial superoxide production and H_2_O_2_ production, we previously reported that DETC (2 mM) augmented the production of mitochondrial superoxide instigated by KI (100 *μ*M) at 2 h and that DETC decreased the H_2_O_2_ production induced by KI [2, 39–41].

Superoxide dismutase transforms superoxide anion into less reactive H_2_O_2_. The iodide has a stimulatory effect on the production of H_2_O_2_ [42]. Ionic iodide is oxidized into its molecular form (I_2_) by thyroid peroxidase [43]. Iodide excess has been related to the progression of thyroid disease in human beings and animal experiments [39, 42–52]. We demonstrated in the present study that the higher the production of superoxide is, the more it is vulnerable to oxidative stress. Ensuing exposure to elevated iodide concentrations, endogenous antioxidant systems are saturated by ROS produced in surplus [1, 43]. Excessive molecular iodide induces apoptosis via a mechanism of free radicals [43, 53]. Mitochondria are the crucial site where ROS is generated [54]. During respiration, mitochondrial complexes I and III generate superoxide. ROS can also be generated by the activation of growth factor receptors, which in turn activate NADPH oxidase that oxidizes NADPH to generate superoxide [55]. In light of literatures and our previous work, we have proposed a chain of events that begin with mitochondrial oxidative stress concluding in apoptosis proceeded by exposure of elevated concentrations of iodide [41]. Keeping mitochondrial ROS at a low level with inhibitors may be beneficial for the management of severe Graves' disease [56]. It is formerly displayed that superoxide anions can selectively trigger apoptosis in umbilical vein endothelial cells in human [57, 58]. It is superoxide anions that regulate the apoptosis instigated by proline oxidase [59]. It has been reported recently that “neutral” TSH receptor antibodies may induce apoptosis by means of stimulating mitochondria ROS production [56].

Besides, we demonstrated that SOD 1000 unit/mL treatment attenuates 100 *μ*M KI-instigated production of mitochondrial superoxide and expression of Prx 3 protein in FRTL cells. SOD is the major antioxidant enzyme that hunts for superoxide anion and the primary antioxidants involved in protecting the mitochondria from oxidative damage [60]. Levels of superoxide anions are kept low in the cell by the enzyme of SOD. It is responsible for the catalyzation reaction that converts superoxide radicals into H_2_O_2_ and molecular oxygen (O_2_). SOD competes with nitric oxide (NO) for superoxide anions; the latter reacts with NO to form peroxynitrite, an inducer of apoptosis. Interestingly, by using triple fluorescence staining, we observed stronger Prx 3 fluorescent intensity than MitoSOX in KI and SOD treatment group, which make the result of decreased LDH release and increased relative viability in this group of treatment reasonable. SOD has been reported to prevent the conversion of NO to peroxynitrite and suppress the apoptosis in cultured rat ovarian follicles, neural cell lines, and transgenic mice [61–63]. Low SOD activity and concentration have been shown in thyroid tissue of patients suffering from endemic goiter who were previously treated with iodized oil injection [64]. In various other thyroid disorders, including thyroid cancer, SOD has also been found to be low [31, 65].

Furthermore, we indicated that SOD inhibitor DETC (2 mM) significantly enhanced KI (100 *μ*M) induced mitochondrial superoxide production and Prx 3 protein expression; the inhibition of endogenous SOD activity led to the results. This supports the report that mitochondrial oxidative stress and cerebral infarction are aggravated in mutant mice without superoxide dismutase [66]. Therefore, our results suggest that SOD 1000 unit/mL in the present study has an important protective role as an antioxidant enzyme in the production of mitochondrial superoxide in elevated concentrations of iodide induced oxidant/antioxidant balance.

We demonstrated that the expression of Prx 3 protein had a substantial increment in the iodide excess-induced oxidative stress in FRTL cells, which was verified by fluorescence immunostaining. We suggest that the increase of Prx 3 expression may be the protective response against high ROS generation, especially H_2_O_2_. The possible explanations are as follows: iodide stimulates the production of H_2_O_2_ [42]. H_2_O_2_ is an important electron acceptor for iodination of iodide and formation of thyroid hormones. The oxidative effect of H_2_O_2_ was dose dependent; 50 *μ*M H_2_O_2_ could instigate cell apoptosis. Rapid cell disturbance occurred due to the toxic effects of H_2_O_2_. Early signs of apoptosis were noticed within the first hour after the onset of exposure to 50–300 *μ*M H_2_O_2_ [42]. We have previously demonstrated a strong increase of superoxide anion production in FRTL cells instigated by elevated concentrations of iodide [2]. Thyrocytes have antioxidant mechanisms, such as SOD and Prxs, that contribute to antioxidative stress [17]. The balance between the production of oxidant species and the antioxidant system is what determines whether a cell dies from ROS-induced apoptosis. MnSOD converted superoxide to H_2_O_2_, which is then metabolized by Prx 3 to water [43]. TSH-induced hydrogen peroxide production and their removal require Prxs in the thyroid cells under normal physiological conditions [67]. When excess H_2_O_2_ or other ROS are produced, Prxs will contribute to limit cellular injuries [17]. It is estimated that Prx 3 is the target for up to 90% of H_2_O_2_ generated in the mitochondrial matrix [68]. Mitochondrial Peroxiredoxins (3 and/or 5) can reduce H_2_O_2_ to water through reducing equivalents provided by thiol-containing proteins [68, 69]. In brain mitochondria, the thioredoxin/Peroxiredoxin (Prx 3 and Prx 5) is the main causative system in the removal of H_2_O_2_ [70]. Findings of increased pyrazole-induced protein carbonylation as well as the formation of 4-HNE adducts and MDA in the livers of Prx 3^−/−^ mice provide enough evidence of its antioxidative role [71].

In addition, we showed significantly decreased expression of Prx 3 protein in KI + SOD group but a significant increased Prx 3 protein expression in KI + DETC group, KI + Rotenone group, and KI + Antimycin A group. This may be explained by the fact that mitochondrial Prxs can compete strongly for hydrogen peroxide in vivo at low levels of hydrogen peroxide, but they are likely to become inefficient at higher levels as the recycling rate becomes a more important factor [20].

The impact that the mitochondrial complex inhibitors have on the KI induced oxidative stress indicated a significant increase of mitochondrial superoxide production, Prx 3 protein expression, LDH release, and decreased relative cell viability in the KI and Rotenone treatment group as well as in the KI and Antimycin A treatment group.

Rotenone is a mitochondrial complex I inhibitor. It is known that Rotenone blocks the electron passage from Fe-S centers of complex I to ubiquinone (CoQ) [72, 73]. Dysfunction of mitochondria caused by Rotenone treatment is related to the release of ROS and activated the glial cells [74]. The impairment of mitochondrial complex I has been suggested in Parkinson's disease. Recent experimental work with Rotenone, a complex I inhibitor, has demonstrated this irregularity [75].

Antimycin A may induce apoptosis and inhibit the electron transport in mitochondrial chain from cytochrome b to cytochrome c1 and lead to elevated ROS generation, and, thereby instigated damage to lipids, DNA, and proteins in cells [76, 77].

Research indicates that nearly complete dysfunction of the mitochondria occurs in the existence of both inhibitors [38]. The inhibitors impair mitochondrial functions via different mechanisms such as the inhibition of cytochrome oxidase by competing with oxygen and nitration of the proteins in the ETC, thus inhibiting their activity [78]. The reduced functioning of ETC results in excessive consumption of mitochondrial GSH (mtGSH) and reduced activity of SOD [79]. Therefore, we suggest that mitochondrial electron transport chain inhibitors, Rotenone and Antimycin A, cause dysfunction of mitochondria which may take effect in iodide excess-induced oxidative stress.

We demonstrated that cleaved caspases 3 and 9 of KI treatment group was significantly increased instigated by KI (100 *μ*M) at 2 h, which could be attenuated by SOD (1000 unit/mL), while they are enhanced by DETC, Rotenone, or Antimycin A treatment. Our findings are consistent with our preceding report, which demonstrated a significant mitochondrial superoxide production with cytochrome c release following elevated concentrations of iodide in FRTL cells [2]. Initiation of caspase-dependent programmed cell death cocured after cytochrome c was released from the mitochondria into the cytoplasm. Procaspase 9 was cleaved after the release of cytochrome c. The activated caspase 9 could mediate the activation of procaspases 3 and 6. Once caspase 3 is activated, it would lead to the cleavage of PARP, which subsequently leads to the cell to undergo apoptosis [80]. Caspases play critical roles in the initiation and execution of apoptosis. ROS is involved in ischemia/reperfusion induced apoptosis [40, 41]; it was demonstrated that the positive cells of cleaved caspase 3 were significantly increased in the ischemia/reperfusion group [81]. The 6-hydroxydopamine- (6-OHDA-) induced oxidative responses are mediated by ROS; it was shown that 6-OHDA (100 *μ*M) could increase the activity of cleaved caspase 3 at 24 h [82].

## 5. Conclusion

**Figure 8:**
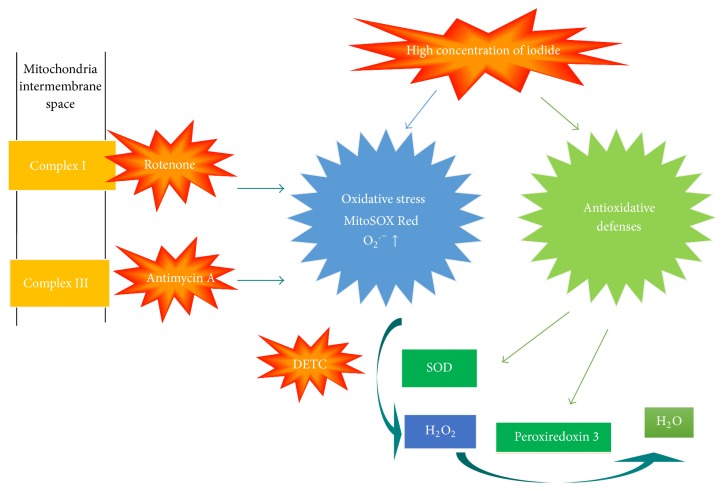
Proposed mechanisms in the present study.

We conclude that mitochondrial respiratory chain complexes I and III inhibitors are potentially involved in the elevated concentrations of iodide instigated oxidative stress and cell apoptosis as well as the protective effect of SOD (Figure 8).

## Abbreviations

FRTL: Fischer rat thyroid cell line
ROS: Reactive oxygen species
ETC: Electron transport chain
SOD: Superoxide dismutase
DETC: Diethyldithiocarbamic acid
Prx 3: Peroxiredoxin 3.
